# Exposure to High Aerial Ammonia Causes Hindgut Dysbiotic Microbiota and Alterations of Microbiota-Derived Metabolites in Growing Pigs

**DOI:** 10.3389/fnut.2021.689818

**Published:** 2021-06-11

**Authors:** Shanlong Tang, Ruqing Zhong, Chang Yin, Dan Su, Jingjing Xie, Liang Chen, Lei Liu, Hongfu Zhang

**Affiliations:** ^1^State Key Laboratory of Animal Nutrition, Institute of Animal Science, Chinese Academy of Agricultural Sciences, Beijing, China; ^2^College of Animal Science and Technology, Qingdao Agricultural University, Qingdao, China

**Keywords:** high ammonia, microbiota, bile acid, short-chain fatty acid, growing pigs

## Abstract

Ammonia, an atmospheric pollutant in the air, jeopardizes immune function, and perturbs metabolism, especially lipid metabolism, in human and animals. The roles of intestinal microbiota and its metabolites in maintaining or regulating immune function and metabolism are irreplaceable. Therefore, this study aimed to investigate how aerial ammonia exposure influences hindgut microbiota and its metabolites in a pig model. Twelve growing pigs were treated with or without aerial ammonia (35 mg/m^3^) for 25 days, and then microbial diversity and microbiota-derived metabolites were measured. The results demonstrated a decreasing trend in leptin (*p* = 0.0898) and reduced high-density lipoprotein cholesterol (HDL-C, *p* = 0.0006) in serum after ammonia exposure. Besides, an upward trend in hyocholic acid (HCA), lithocholic acid (LCA), hyodeoxycholic acid (HDCA) (*p* < 0.1); a downward trend in tauro-deoxycholic acid (TDCA, *p* < 0.1); and a reduced tauro-HDCA (THDCA, *p* < 0.05) level were found in the serum bile acid (BA) profiles after ammonia exposure. Ammonia exposure notably raised microbial alpha-diversity with higher Sobs, Shannon, or ACE index in the cecum or colon and the Chao index in the cecum (*p* < 0.05) and clearly exhibited a distinct microbial cluster in hindgut indicated by principal coordinate analysis (*p* < 0.01), indicating that ammonia exposure induced alterations of microbial community structure and composition in the hindgut. Further analysis displayed that ammonia exposure increased the number of potentially harmful bacteria, such as *Negativibacillus, Alloprevotella*, or *Lachnospira*, and decreased the number of beneficial bacteria, such as *Akkermansia* or *Clostridium_sensu_stricto_1*, in the hindgut (FDR < 0.05). Analysis of microbiota-derived metabolites in the hindgut showed that ammonia exposure increased acetate and decreased isobutyrate or isovalerate in the cecum or colon, respectively (*p* < 0.05). Unlike the alteration of serum BA profiles, cecal BA data showed that high ammonia exposure had a downward trend in cholic acid (CA), HCA, and LCA (*p* < 0.1); a downward trend in deoxycholic acid (DCA) and HDCA (*p* < 0.05); and an upward trend in glycol-chenodeoxycholic acid (GCDCA, *p* < 0.05). Mantel test and correlation analysis revealed associations between microbiota-derived metabolites and ammonia exposure-responsive cecal bacteria. Collectively, the findings illustrated that high ammonia exposure induced the dysbiotic microbiota in the hindgut, thereby affecting the production of microbiota-derived short-chain fatty acids and BAs, which play a pivotal role in the modulation of host systematic metabolism.

## Introduction

Ammonia (NH_3_), the sole alkaline gas in the atmosphere, is the predominant source of active nitrogen. The primary sources of aerial ammonia are agricultural production, such as emission from animal husbandry and release of ammonia-based fertilizer (1, 2), and industrial production (e.g., chemical pant), land or sea release. Human activities (e.g., automobiles and airplane emissions) also give off ammonia (3). There is increasing attention to ammonia release over the past few decades, because of it having adverse influences on animal and human health. Besides, pernicious effects of aerial ammonia on the formation of atmospheric particles and reduction of air visibility have been reported (4, 5). Numerous studies have demonstrated that atmospheric ammonia has hazardous effects on many organs of animals, causing cardiac autophagy or liver apoptosis *via* the mitochondrial pathway or the PETEN/AKT/mTOR pathway (6–8), leading to respiratory tract infection and inflammation response (9), bringing about intestinal microvilli deficiency (10) and microbial disturbance in the small intestine (11), giving rise to dysfunction of immune organs (12, 13).

Previous studies in our laboratory have proved metabolic disorders induced by aerial ammonia exposure in animals. Ammonia exposure modulated the distribution of body fat in broilers by regulating the transcripts of lipid metabolism-related enzymes in the liver or breast muscle (14, 15). The previous results exhibited that high ammonia exposure disordered lipid metabolism *via* activation of the mTOR pathway, consequently upregulating genes involved in lipogenesis and downregulating lipolysis genes in the muscle of growing pigs (16), and impaired the branched-chain amino acid (BCAA) catabolism by suppressing the expression of BCAA catabolism-related enzymes (unpublished data). Attractively, the type of skeletal myofiber, especially increased myosin heavy chain (MyHC) *IIx* or decreased MyHC *I*, was notably altered after aerial ammonia exposure, which indicated that the metabolic type of skeletal muscle changed from oxidative to glycolytic type (16, 17). However, the specific mechanism that aerial ammonia jeopardizes animal health, causing metabolic disorder, is still unclear.

Microbiota is a crucial “microbial organ” of mammals, and is closely related to many physiological functions, such as metabolism, immunity, and nutrition. Recent studies have displayed that microbiota plays a momentous role in the function and metabolism of skeletal muscles (18–20). The absence of gut microbiota induces muscle mass loss and causes metabolic disturbance containing BCAA dysbolism and alteration of muscular myofibers or glucose homeostasis (19, 20). These results caused by the absence of gut microbiota are similar to those induced by aerial ammonia exposure, indicating that the role of gut microbiota in muscular dysbolism caused by ammonia exposure is irreplaceable. Besides, there is a wide array of evidence that microbial metabolites, such as short-chain fatty acid (SCFA), bile acid (BA), microbial tryptophan catabolites, and succinate, are pivotal inducers that regulate host metabolism and inflammatory response (21). However, it is still unclear whether or how ammonia exposure affects the composition of gut microbiota and its metabolites. Therefore, this study aimed to investigate the impacts of aerial ammonia on the constitution of gut microbiota (in the cecum and colon) and its metabolites [SCFA, BA, and amino acid (AA)].

## Materials and Methods

### Animal Experimental Ethics

All procedures performed in this study were reviewed and approved by the Experimental Animal Welfare and Ethical Committee of the Institute of Animal Science of the Chinese Academy of Agricultural Sciences (IAS2017-2). Minimum numbers of pigs were used in an effort to minimize stress during handling.

### Animals and Exposure Conditions

A total of twelve 70-day-old pigs (Yorkshire × Landrace, 20.27 ± 0.36 kg) purchased from a commercial pig farm (Beijing Breeding Pig Co., Ltd., Beijing, China) were randomly distributed into two groups, the control and high ammonia groups. All the pigs were individually penned, and each group was maintained in a separate controlled environment chamber. The pigs in the ammonia chamber were exposed to 35 mg/m^3^ ammonia for 25 days, while pigs in the control group were housed in another chamber without ammonia during the experimental period. ToxiRAE Pro Ammonia (NH_3_) Detector (RAE systems, San Jose, CA, USA) was used to monitor ammonia concentration in the chamber, and ammonia was sent into the chamber *via* a ventilation system before being mixed with air. Over the 25-day experimental period, all pigs had free access to clean water and consumed commercial feed (Supplementary Table 1) that was equal to 4–5% of body weight (BW) per day. The BW of each pig was taken weekly, and all animals were allowed to adapt to the chamber for 7 days under control group conditions before the start of the experiment.

### Collecting Samples

At the end of the experiment, blood samples were acquired from the jugular vein *via* a sterilized syringe before the pigs were sacrificed by electric stunning (Xingye Butchery Machinery Co. Ltd., Changde, China). Then, the blood was centrifuged at 3,000 rpm for 15 min to obtain serum after 3 h incubation at room temperature. The serum was aliquoted and stored at −80°C for BA quantification or other metabolite analysis. Sections of the cecum and proximate colon were *in situ* ligated before the whole intestine was removed from the abdominal cavity. Then, the digesta in the cecum and proximal colon was aseptically collected in 2-ml sterile tubes, immediately frozen in liquid nitrogen, and stored at −80°C for sequencing of microbial 16S genes and analysis for SCFA and BA quantification.

### Serum Metabolites

Serum leptin and adiponectin were measured by ELISA for antigen detection using commercial assay kits (Cat # KAP2281 for leptin, Cat # KAPME09 for adiponectin) from Beijing North Institute of Biological Technology (Beijing, China). According to the instruction of the manufacturer, the concentrations of high-density lipoprotein cholesterol (HDL-C, Cat # A112-2-1), low-density lipoprotein cholesterol (LDL-C, Cat # A113-2-1), very low-density lipoprotein (VLDL, Cat # H249), alanine aminotransferase (ALT, Cat # C009-2-1), and aspartate aminotransferase (AST, Cat # C010-2-1) in the serum were detected *via* commercial assay kits purchased from Nanjing Jiancheng Bioengineering Institute (Nanjing, China).

### Quantification of Bile Acids in Serum and Intestinal Digesta

The BA in the serum was extracted according to the methods described by Fang et al. (22). Briefly, 200 μl of the serum was added into an equal amount of pre-cold sodium acetate (50 mM, pH 5.6) and triple ethanol (chromatography grade), and then the mixture was vortexed for 2 min to mix evenly. After centrifugation at 20,000 g for 20 min, the supernatant was diluted five times with a sodium acetate buffer and applied to a Bond Elute C18 cartridge (500 mg/6 ml, Varian, Harbor City, CA, USA) pre-activated by 5 ml methanol. The cartridge was then washed with 25% ethanol, and the BA was eluted with 5 ml methanol. The residue was dissolved in 1 ml methanol after the solvent was evaporated with nitrogen gas and finally passed through a 0.45-μm Milled-LG filter (Millipore, Billerica, MA, USA) for BA analysis. The BA in the intestinal digesta was extracted according to the methods described by Fang et al. (22). Approximate 50–80 mg lyophilized digesta in cecum was suspended in a mixture of pre-cold sodium acetate buffer (50 mM, pH 5.6) and ethanol, and then the same method was used as described above.

The BA in the serum and intestinal digesta was profiled with a Waters Xevo TQ-S LC/MS mass spectrometer (Waters, Milford, MA, USA) equipped with an ESI source and the assay condition used in the previous report by Fang et al. (22). Briefly, a 10-μl filtrate was injected into a ZORBAX Eclipse plus C18 column (95 Å, 1.8 μm, 2.1 × 100 mm) from Agilent (Santa Clara, CA, United States) to separate the BA. The mobile phases consisted of 5% acetonitrile and 0.1% formic acid (mobile phase A) and 95% acetonitrile and 0.1% formic acid (mobile phases B). The gradient for BA elution was gradually changed at a total flow rate of 0.4 ml/min as follows: mobile phase A:B (9:1, v/v) from 0 to 1 min, mobile phase A:B (7:3, v/v) from 1 to 1.5 min, mobile phase A:B (2:3, v/v) from 1.5 to 5.5 min, and mobile phase A:B (9:1, v/v) from 5.5 to 7 min. The spray voltage and vaporizer temperature were set at 2.91 kV and 500°C, respectively. The gas flow was set at 550 L/h. A total of 18 BA standards were purchased from Sigma-Aldrich (Merck KGaA, Darmstadt, Germany). The quantification of each BA was based upon the series dilutions of available standards, and good linearity was confirmed.

### Quantification of Short-Chain Fatty Acids in Intestinal Digesta

To extract SCFA, about 0.5 g wet digesta was thoroughly mixed with 5 ml ultrapure water, then shocked for 30 min to mix evenly, and finally incubated at 4°C for 24 h. After centrifugation at 12,000 rpm for 20 min, the supernatant was mixed with 25% metaphosphoric acid at a ratio of 9:1, vortexed, and incubated at room temperature for 4 h. Then, the mixture was passed through a 0.45-μm Milled-LG filter (Millipore, Billerica, MA, USA) and subjected to SCFA analysis.

The Agilent 7890N gas chromatograph (Agilent, Santa Clara, CA, USA) was utilized to detect the SCFA in the samples. Briefly, a 2-μl sample was injected (split ratio 1:50) into the gas chromatograph equipped with a DB-FFAP column (15 m × 0.32 mm × 0.25 μm). The initial oven temperature was set at 100°C and then raised to 120°C at 2°C/min held at 120°C for 10 min. Nitrogen served as the carrier gas at a constant flow rate of 0.8 ml/min, and the constant pressure was 21.8 kPa. The injector and detector temperatures were 250 and 280°C, respectively. Individual SCFAs were identified by comparing their retention times with those in the standard mix of SCFA standards purchased from Sigma-Aldrich (Merck KGaA, Darmstadt, Germany).

### DNA Extraction, Amplification, and Sequencing

Total bacterial DNA was extracted from the intestinal digesta using the EZNATM Soil DNA kit (D5625-02, Omega Bio-Tek Inc., Norcross, GA, USA) according to the instructions of the manufacturer. The V3-V4 hypervariable regions of the bacterial 16S rDNA were amplified by a two-step PCR method using primers 338F (5′-ACTCCTRCGGGAGGCAGCAG-3′) and 806R (5′-GGACTACCVGGGTATCTAAT-3′) with unique 8-bp barcodes to facilitate multiplexing, and sequencing was carried out with an Illumina sequencing platform using Miseq PE300.

### Data Analysis and Statistical Test

Student's *t*-test of the data on serum metabolites (ALT, AST, LDL-C, VLDL, HDL-C, leptin, adiponectin, and BAs), microbial metabolites (intestinal BAs and SCFAs), and bacterial alpha-diversity indices (Sobs, Shannon, ACE, and Chao) was performed using the JMP software (JMP^®^ version 10.0.0, SAS Institute, Cary, NC, USA) for Windows. *P* < 0.05 was regarded as statistically significant, while 0.05 < *p* < 0.1 was set as significant trend.

Raw data obtained from gut microbiota were processed using the free online platform of Majorbio I-Sanger Cloud Platform (www.i-sanger.com), and redundant sequences were filtered. UPARSE (version 7.1, http://drive5.com/uparse/) was used to cluster operational taxonomic units (OTUs) at 97% similarity cutoff, and each presentative OTU was mapped to the Silva 138 database by RDP classifier (http://rdp.cme.msu.edu/) at a confidence threshold of 0.7. The principal coordinate analysis, triplot of redundancy analysis (RDA), and network for correlation analysis were employed using Majorbio I-Sanger Cloud Platform, and the Spearman's or Mantel's correlation analysis was applied using the ggcor R package. The significant difference between the control group and the ammonia group at genus level was tested by the DESeq2 method (MicrobiomeAnalyst, https://www.microbiomeanalyst.ca/) with corrected *p*-value (FDR) < 0.05.

## Results

### Serum Metabolites Related to Lipids and Amino Acids

**Figure 1:**
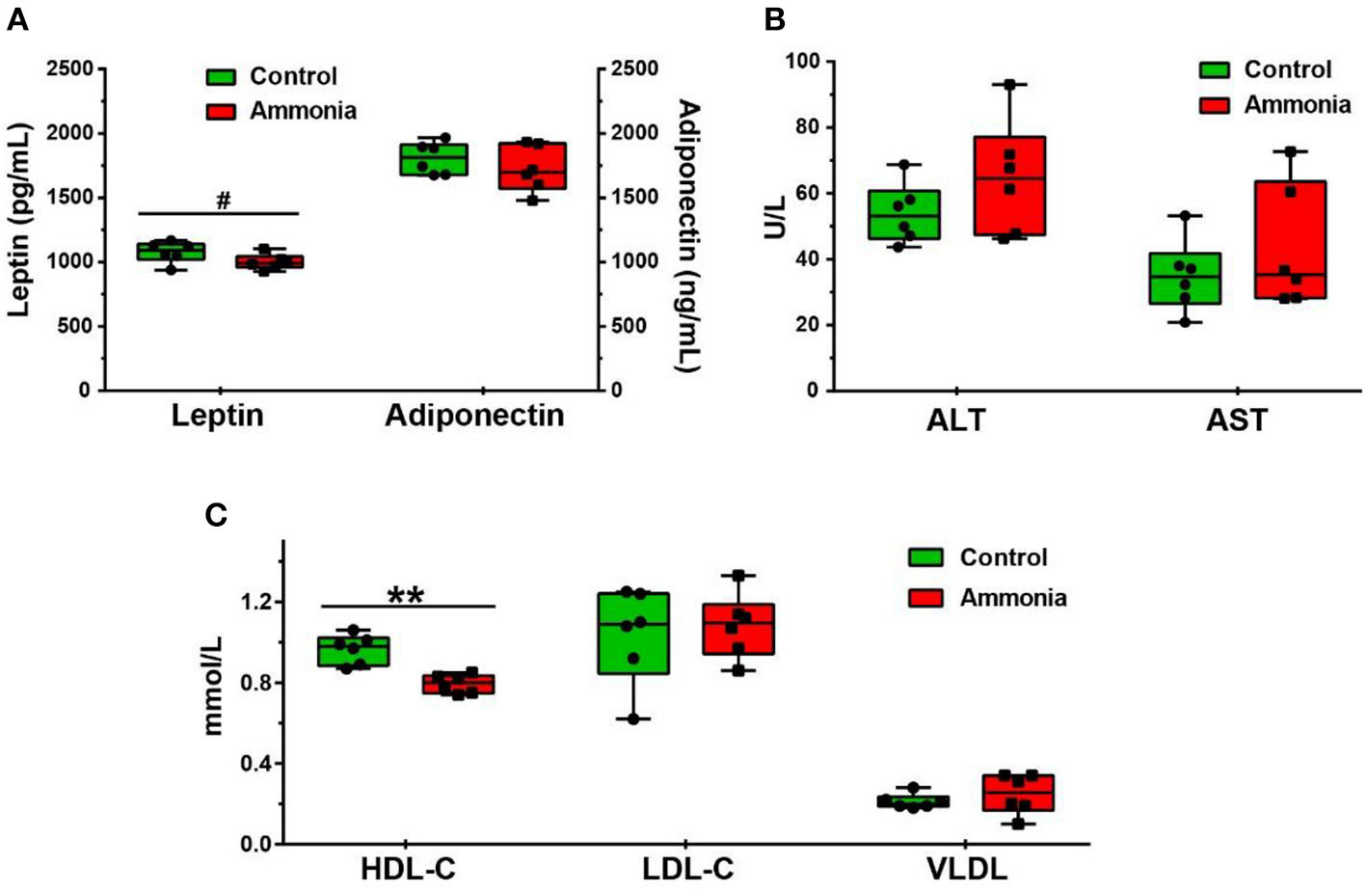
The level of serum metabolites after atmospheric ammonia exposure. The contents of serum leptin and adiponectin after high ammonia exposure. **(A)** The level of ALT and AST in serum after high ammonia exposure. **(B)** The alteration of HDL-C, LDL-C, and VLDL in serum after ammonia exposure. **(C)** Data are expressed as min to max showing all points (*n* = 6), ^#^*p* < 0.1 and **p < 0.01.

Although no changes in serum adiponectin, ALT, AST, LDL-C, and VLDL were observed in pigs exposed to high ammonia (*p* > 0.05, Figures 1A–C, Supplementary Table 2), serum HDL-C was diminished (*p* = 0.0006, Figure 1C, Supplementary Table 2), and serum leptin had a reduced trend (*p* = 0.0898, Figure 1A, Supplementary Table 2) after high ammonia exposure.

The results of other serum metabolites and free AAs, as described by Tang et al. (16), demonstrated that high ammonia exposure increased the concentration of serum total triglycerides (TG, *p* = 0.0294, Supplementary Table 2) and ApoB (*p* = 0.0061, Supplementary Table 2).Compared with the control pigs, the serum BCAA [leucine (Leu), *p* < 0.0001; isoleucine (Ile), *p* = 0.0016; valine (Val), *p* = 0.0047] and aromatic AA [tyrosine (Tyr), *p* < 0.0001; phenylalanine (Phe), *p* = 0.0002] were also notably increased in pigs exposed to high atmospheric ammonia (Supplementary Table 2). A previous study found that no alterations in feed intake, BW, and body weight gain (BWG) were observed in high ammonia exposed pigs (*p* > 0.05, Supplementary Figures 1A–C) (16).

### Serum Bile Acid Profiles

**Figure 2:**
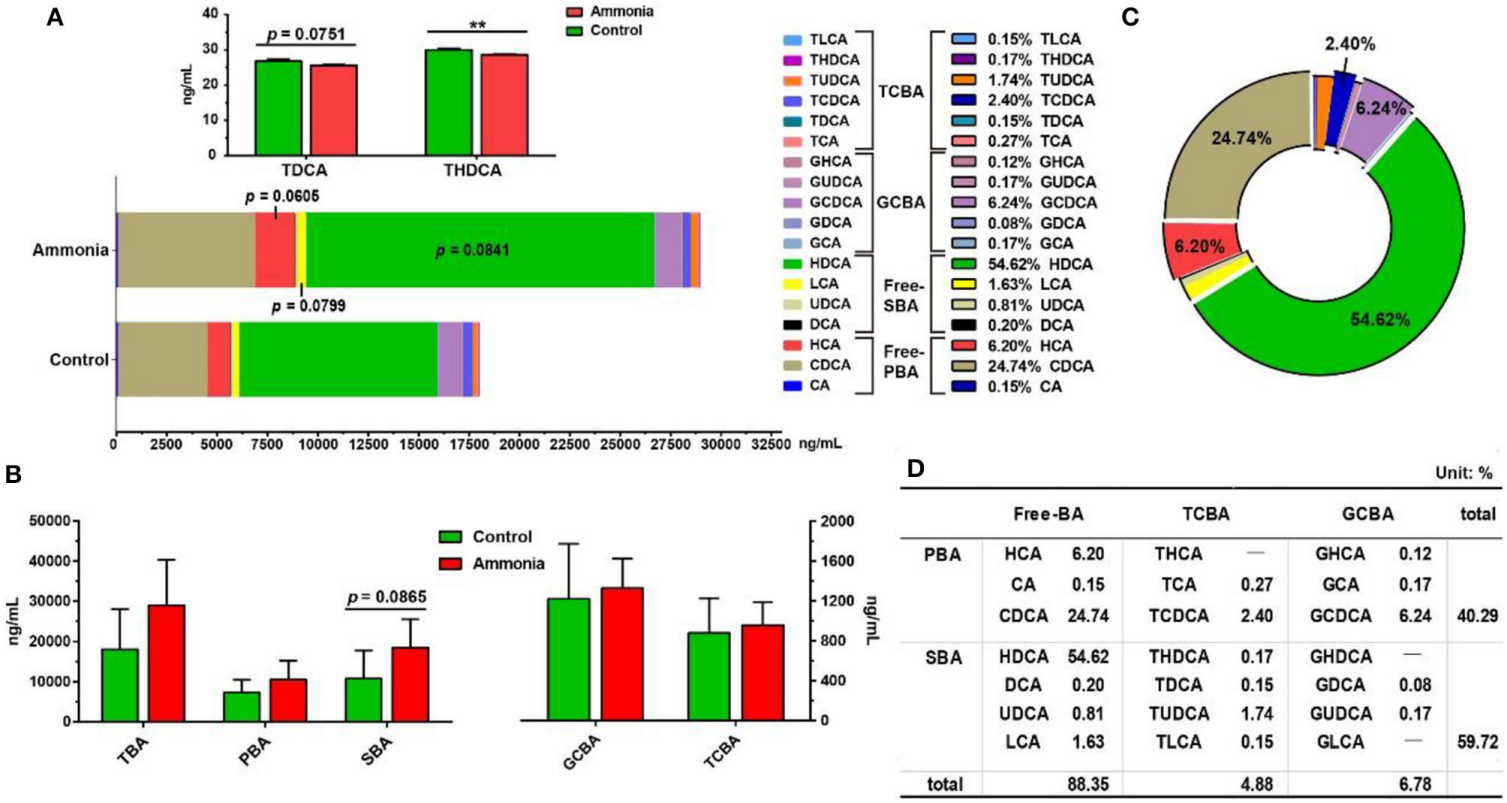
The alteration in serum BA profiles caused by ammonia exposure. The changes in **(A)** each BA content and **(B)** each type of BA content in the serum of pigs exposed to high ammonia; **(C)** the compositions of each BA and **(D)** each type of BA in the serum of control pigs. Data are expressed as mean value or mean ± SE (*n* = 6), **p < 0.01.

In the growing pig serum, ~88% of the BA existed in free form (Figure 2D). The most abundant BA was hyodeoxycholic acid (HDCA, 54.62%) or chenodeoxycholic acid (CDCA, 24.74%) in the secondary BAs (SBAs) or primary BAs (PBAs), respectively, which consisted of ~97% of total serum BAs with glyco-CDCA (GCDCA, 6.24%), hyocholic acid (HCA, 6.20%), tauro-CDCA (TCDCA, 2.4%), tauro-ursodeoxycholic acid (TUDCA, 1.74%), and lithocholic acid (LCA, 1.6%) (Figures 2C,D). Among them, serum HCA (*p* = 0.0605), LCA (*p* = 0.0799) and HDCA (*p* = 0.0841) had an upward trend in pigs exposed to high ambient ammonia, while serum tauro-deoxycholic acid (TDCA, *p* = 0.0751) had a downward trend. and tauro-HDCA (THDCA, *p* = 0.0050) notably decreased in pigs exposed to high ammonia (Figure 2A; Supplementary Table 2). Despite higher differences from a trend point of view, no significant difference was observed in serum total BA (TBA) and PBA (*p* > 0.05, Figure 2B; Supplementary Table 2), and high ammonia exposure tended to increase serum SBA (*p* = 0.0865, Figure 2B; Supplementary Table 2).

### Global Assessments for Sequencing Data

After data trimming and quality control, a total of 809,102 sequences from the cecum digesta and 690,575 sequences from the colon digesta were acquired with the number of sequences ranging from 38,754 to 73,486 per sample. The filtered 539,136 or 343,896 sequences from the cecum or the colon digesta, based on the normalized depth of 44,928 or 28,658 reads per sample, were clustered into 634 or 807 OTUs for all the samples at a 97% sequence similarity value, and were further clustered into 190 or 197 genera, 87 or 82 families, 53 or 51 orders, 25 or 25 classes, and 15 or 16 phyla. Most of the microbial diversity and bacterial communities in the cecum or the colon digesta samples had been sufficiently captured, indicated by good coverage (>0.999) and rarefaction curves (Supplementary Figure 2).

### Variation in Alpha and Beta Diversities of Gut Microbiota

**Figure 3:**
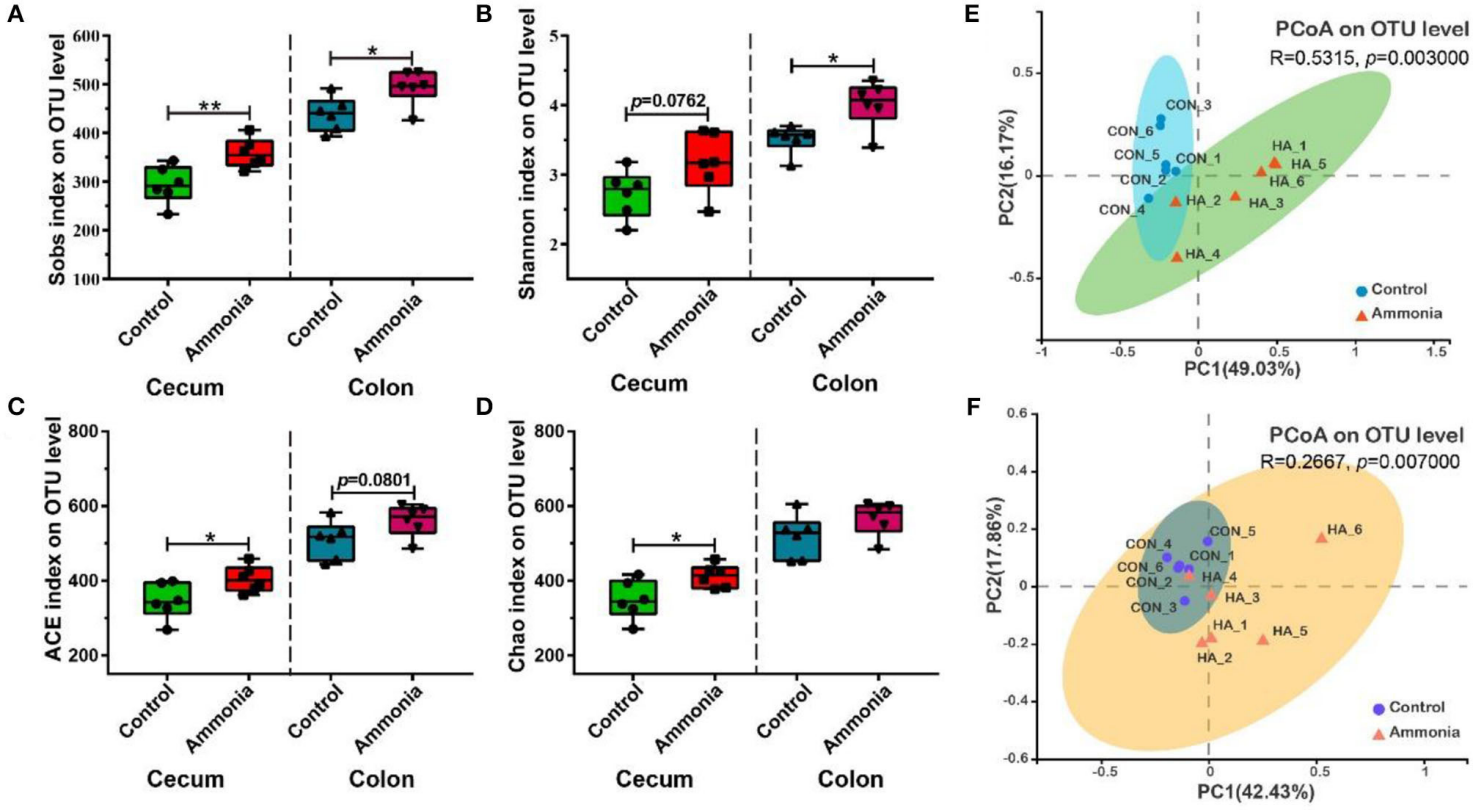
Alpha and beta diversities of the microbial community in the cecum or colon digesta after ammonia exposure. **(A)** The Sobs, **(B)** Shannon, **(C)** ACE, and Chao indexes in the cecum or colon among two groups; PCoA (OTU level) of community membership based on the Bray–Curtis distance and ANOSIM test in **(E)** cecum and **(F)** colon. Data are expressed as min to max showing all points (*n* = 6), *p < 0.05, and **p < 0.01.

Compared with the control group pigs, the ammonia-exposed pigs exhibited higher Sobs (*p* = 0.009, Figure 3A), ACE (*p* = 0.0354, Figure 3C), and Chao indexes (*p* = 0.0268, Figure 3D) and showed a higher trend in the Shannon index (*p* = 0.0762, Figure 3B) in the cecum digesta at the OTU level. In the colon digesta, although no difference in the Chao index was observed (*p* > 0.05, Figure 3D), the Sobs (*p* = 0.0238, Figure 3A) and Shannon indexes (*p* = 0.0115, Figure 3B) were markedly greater and the ACE index (*p* = 0.0801, Figure 3C) showed an upward trend in the ammonia-exposed pigs at the OTU level. The principal coordinates analysis (PCoA) based on Bray–Curtis distance and ANOSIM test revealed that beta-diversity shifted due to ambient ammonia exposure and notable differences were observed in the cecum and colon at the OTU level (cecum R = 0.5315, *p* = 0.003, Figure 3E; colon R = 0.2667, *p* = 0.007, Figure 3F).

### Alteration of Specific Gut Microbiota

**Figure 4:**
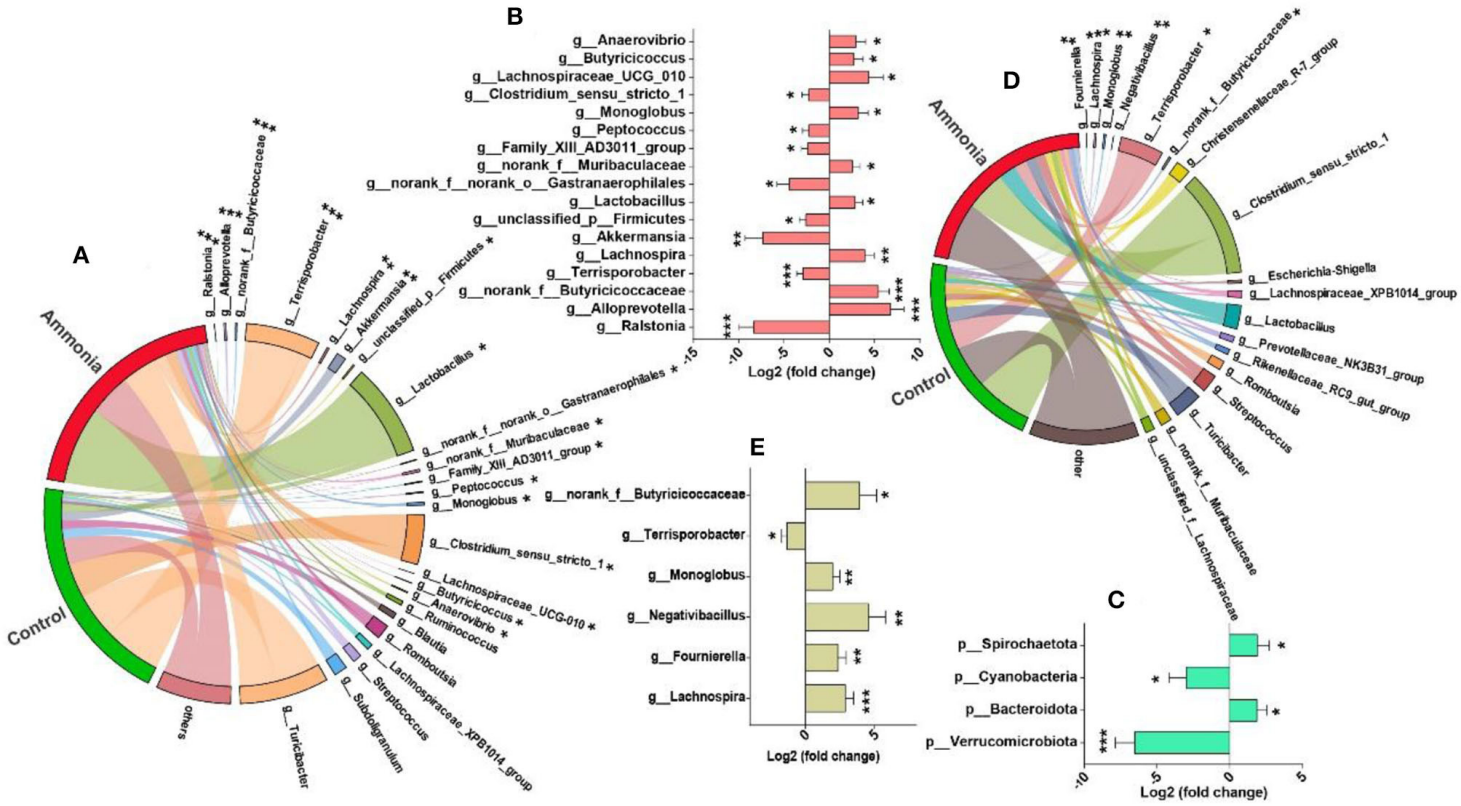
Differentially abundant genera from the cecum or colon after ammonia exposure. The mainly enriched genera (including differentially abundant genera) from **(A)** the cecum or **(D)** colon in control group vs. ammonia group; the differentially abundant **(B)** genera or **(C)** phyla from the cecum and the differentially abundant **(E)** genera from the colon after high ammonia exposure. *FDR < 0.05, **FDR < 0.01, and ***FDR < 0.001, respectively.

A total of 17 microbiotas were identified at genus level in the cecum digesta of the pigs exposed to high ammonia, which included eight downregulated genera (*Ralstonia, Akkermansia, Gastranaerophilales, Terrisporobacter, unclassified_p__Firmicutes, Family_XIII_AD3011_group, Peptococcus*, and *Clostridium_sensu_stricto_1*) and nine upregulated genera (*norank_f__Muribaculaceae, Butyricicoccus, Lactobacillus, Anaerovibrio, Monoglobus, Lachnospira, Lachnospiraceae_UCG_010, norank_f__Butyricicoccaceae*, and *Alloprevotella*) (FDR < 0.05, Figures 4A,B). Besides, four microbiotas (increased *Bacteroidota* and *Spirochaetota*; decreased *Verrucomicrobiota* and *Cyanobacteria*) were notably altered at phylum level in the cecum digesta after ammonia exposure (FDR < 0.05, Figure 4C). In the colon digesta, high ammonia exposure increased genera *Lachnospira, Fournierella, Negativibacillus, Monoglobus, Butyricicoccaceae*, and decreased genus *Terrisporobacter* (FDR < 0.05, Figures 4D,E).

### Gut Short-Chain Fatty Acid and Bile Acid Production

**Figure 5:**
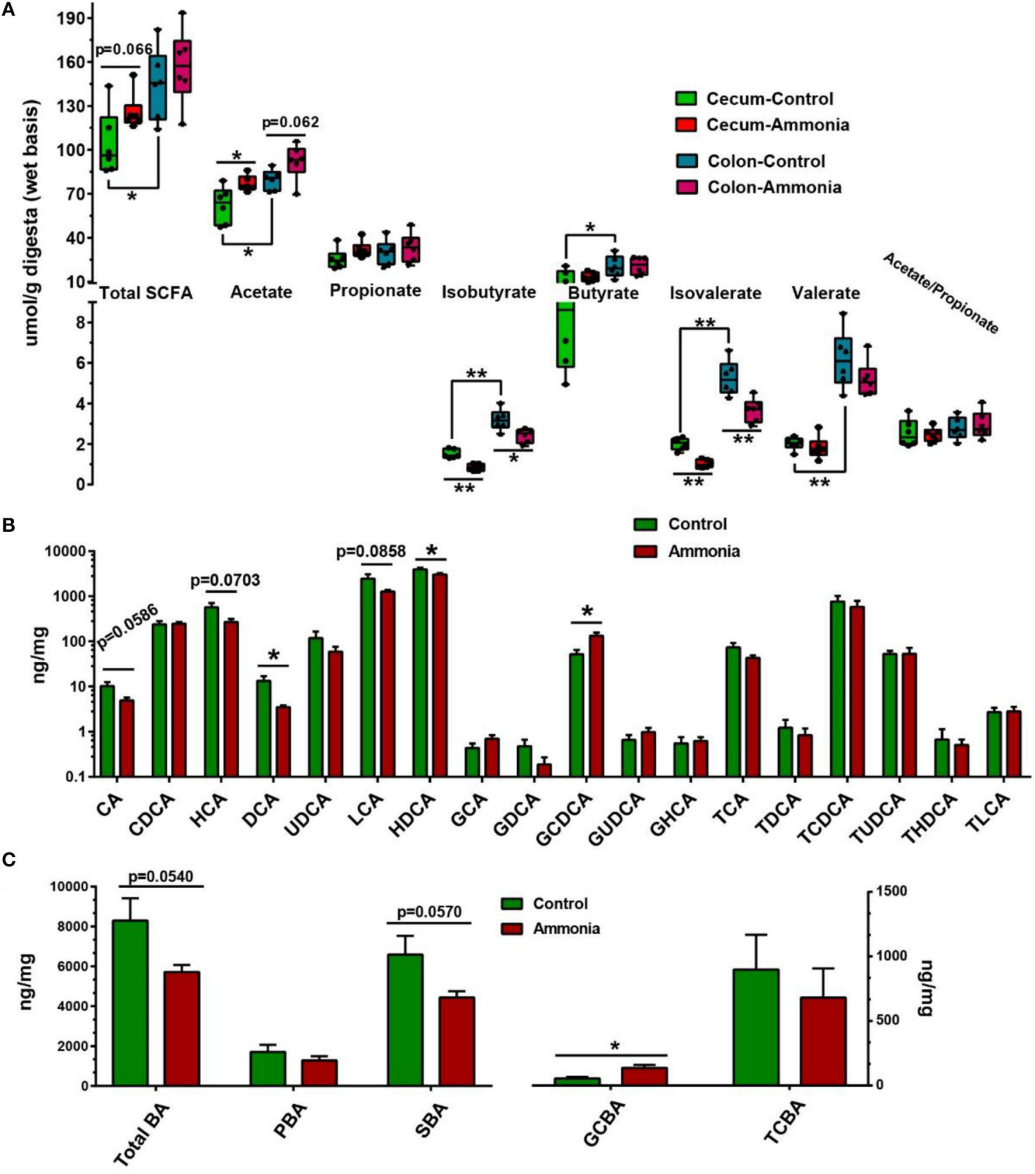
SCFA and BA production from the cecum or colon digesta in pigs exposed to ammonia or not. **(A)** Changes in the SCFA profile of the cecum or colon after ammonia exposure; **(B)** changes in each BA content; and **(C)** each type of BA content in the cecum of pigs exposed to high ammonia. Data are expressed as min to max showing all points or mean ± SE (*n* = 6), *p < 0.05 and **p < 0.01.

Based on microbiota alteration, further investigation of SCFA concentration in the cecum or colon was completed and shown in Figure 5A and Supplementary Table 3. In the cecum digesta, ammonia-exposed pigs exhibited lower concentration of isobutyrate (*p* = 0.0002) and isovalerate (*p* < 0.0001), while the higher concentration of acetate (*p* = 0.0243) and increased trend in total SCFA (*p* = 0.0657) were remarkably observed in the pigs exposed to ammonia. Compared with cecum SCFA, the colon SCFA profiles in either pigs exposed to ammonia or pigs not exposed to ammonia had similar alteration. In the colon digesta, high ammonia exposure decreased the content of isobutyrate (*p* = 0.0115) and isovalerate (*p* = 0.0035) and had a trend to increase acetate concentration (*p* = 0.0624). Besides, the contents of total SCFA, acetate, isobutyrate, butyrate, isovalerate, and valerate in the cecum were higher than those in colon.

Further investigation of BA concentration in the cecum was also finished. Among them, cecal cholic acid (CA, *p* = 0.0586), HCA (*p* = 0.0703), and LCA (*p* = 0.0858) had a downward trend, and deoxycholic acid (DCA *p* = 0.0262) and HDCA (*p* = 0.0437) dramatically decreased in the ammonia-exposed pigs, while cecal GCDCA (*p* = 0.0128) notably increased in the ammonia-exposed pigs (Figure 5B; Supplementary Table 3). Besides, there also was a downward trend in cecal TBA (*p* = 0.0540) and SBA (*p* = 0.057) after ammonia exposure, and high ammonia exposure significantly increased cecal glycine-conjugated BA (GCBA, *p* = 0.0139) (Figure 5C; Supplementary Table 2).

### Microbiota-Metabolites Correlation

**Figure 6:**
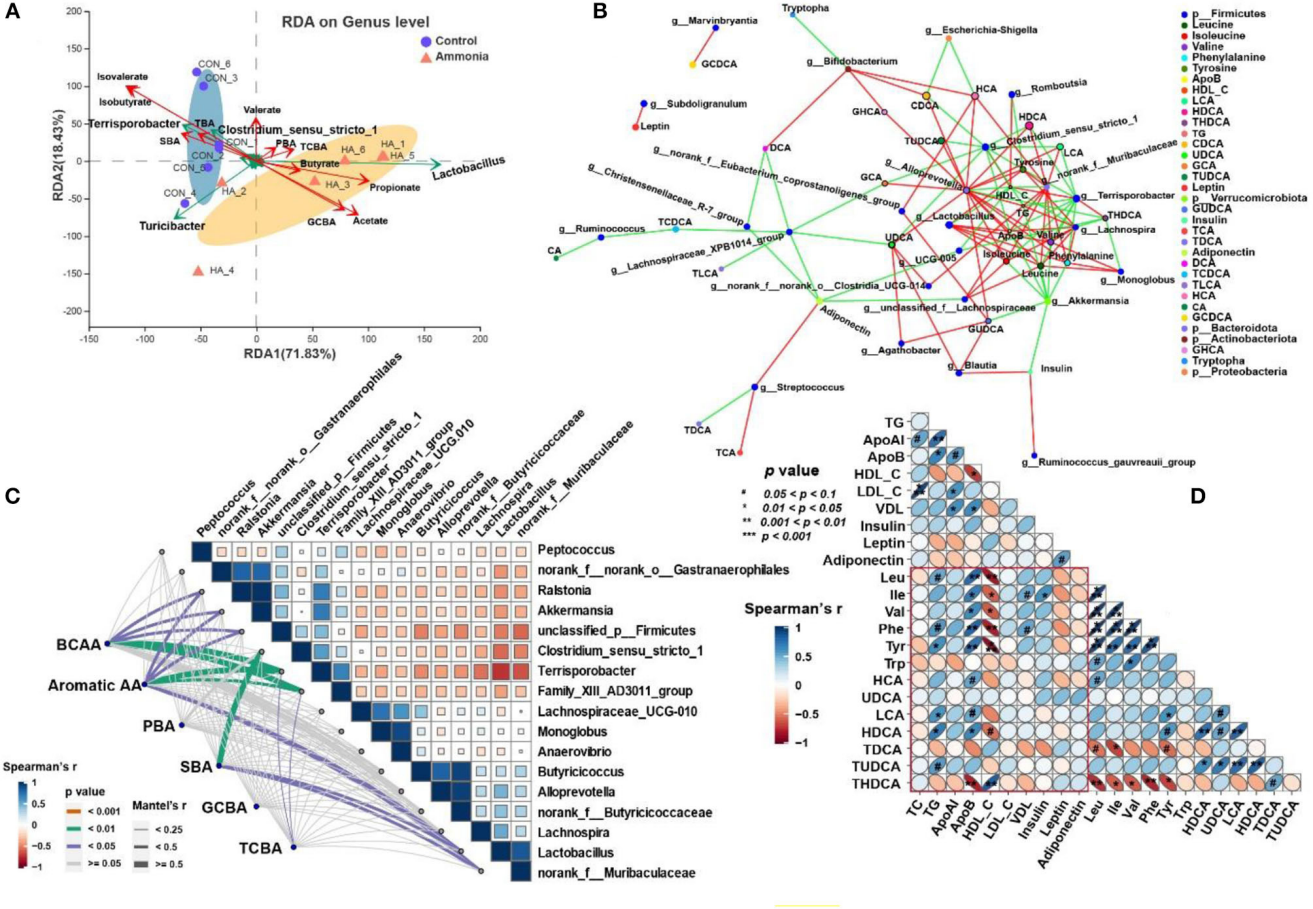
Microbiota–metabolite correlation. **(A)** Triplot of RDA of the cecal microbial composition at genus level relative to cecal BAs and SCFAs. Microbiota from the control and ammonia groups is indicated by blue circles and orange triangles, respectively. Constrained explanatory variables (cecal TBA, PBA, SBA, GCBA, TCBA, and SCFA) are indicated by red arrows. Responding taxa are indicated by green arrows, and only those with higher fit in the ordination plot are labeled. The first (71.83% interpretation) and second coordinates (18.43% interpretation) are plotted. **(B)** A network for correlation analysis among the top 50 relative abundance of cecal bacterial genera, serum BA, and AA using relevant networking. Only correlations with Spearman's coefficient >0.5 and *p* < 0.05 are shown. The different color of nodes indicates various targets as demonstrated in legend. The line color represents correlation, and red and green represent positive and negative correlation, respectively. The more lines there are, the more connected the nodes are. **(C)** Pairwise comparisons of different cecal genera are demonstrated with a color gradient denoting Spearman's correlation coefficient. BCAA, aromatic AA, PBA, SBA, TCBA, and GCBA are related to each microbial genus by Mantel's correlation analysis. Edge width corresponds to the Mantel's r statistic for the corresponding distance correlation, and edge color denotes statistical significance. **(D)** Half Spearman's correlation matrix of serum BCAA (Leu, Ile, and Val), Aromatic AA (Phe, Tyr, and Trp), serum BA, and lipid parameters. The red and blue ellipses represent negative and positive correlations, respectively, and the width of ellipses represents strength of correlation (narrow ellipses mean stronger correlation).

The triplot of RDA was shown in Figure 6A and revealed that the cecal samples from the control or the ammonia group were separated at the first constrained axis. *Clostridium_sensu_strictio_1, Terrisporobacter*, and *Turicibacter* were positively correlated with SBA, TBA, isovalerate, and isobutyrate in cecum chyme, while *Lactobacillus* was positively correlated with GCBA, taurine-conjugated BA (TCBA), acetate, butyrate, and propionate in cecum chyme. To further investigate the relationship between cecum bacteria and serum metabolites, the relevance network association analysis and Spearman or Mantel correlation analysis were established by the abundance of cecal genera, serum BCAA or aromatic AA, and lipid-related metabolites. The network analysis by Spearman correlation revealed that cecal *Akkermansia* and *Terrisporobacter* abundance were negatively associated with each BCAA in the serum, while *Alloprevotella, norank_f__Muribaculaceae, Lactobacillus, Monoglobus*, and *Lachnospira* abundance were positively associated with each serum BCAA (Spearman's r > 0.5, *p* < 0.05, Figure 6B). Because of the similarly altered trend between each BCAA and aromatic AA, the same genera and relationship were observed in each aromatic AA by network analysis (Spearman's r > 0.5, *p* < 0.05, Figure 6B). In addition, the Mantel correlation analysis demonstrated that a significant correlation was observed between five genera (*Ralstonia, Akkermansia, unclassified_p__Firmicutes, Terrisporobacter*, and *Family_XIII_AD3011_group*) and total BCAA (Mantel's r > 0.25, *p* < 0.05, Figure 6C). Apart from the same five genera, there were still two genera (*Lactobacillus* and *norank_f__Muribaculaceae*) which had a significant correlation with total aromatic AA by Mantel correlation analysis (Mantel's r > 0.25, *p* < 0.05, Figure 6C). For serum BAs, only serum SBA had a significant correlation with microorganisms (*Lactobacillus, norank_f__Muribaculaceae, Clostridium_sensu_stricto_1, Terrisporobacter, Alloprevotella, Akkermansia, Monoglobus*, and *Lachnospira*; r > 0.25, *p* < 0.05, Figures 6B,C). Spearman's correlation analysis showed that there was a strong correlation between BCAAs (Leu, Ile, Val), aromatic AAs (Tyr, Phe) or partial BAs (HCA, LCA, HDCA, TUDCA, THDCA), and lipid-related metabolites (TG, ApoB, HDL-C, VDL; *p* < 0.05, Figure 6D).

## Discussion

Increasing aerial ammonia, the most infamous atmospheric pollutant, has attracted much attention recently, owing to its adverse impacts on animal and human metabolic states. The serum lipid-related metabolites may reflect the overall metabolic state to a certain extent. Elevated serums, TG and ApoB, were observed in the previous study after high ammonia exposure (16). Evidence is given that a high level of ApoB is a superior indicator of vascular heart disease driving physiology than either total cholesterol (TC) or LDL-C (23). This study found no alteration in LDL-C, but there was a remarkable decline in HDL-C level after high ammonia exposure. HDL-C exhibited extraordinary anti-inflammation and antioxidant ability because of the existence of multiple antioxidant enzymes and the ability to neutralize bacterial lipopolysaccharide (LPS) (24). The low HDL-C level was usually associated with high triglyceride as the precursor of dysmetabolic events, such as insulin resistance. The alterations in lipid-related metabolites, including TG, HDL-C, and ApoB, caused by ammonia exposure reflected the transformation of metabolic state, which was closely related to the serum microbiota-derived metabolites (such as BCAA, aromatic AA, HCA, LCA, HDCA, and THDCA) indicated by the Spearman's correlation analysis.

Lipid metabolism of skeletal muscle and metabolic state were altered in pigs after high aerial ammonia exposure in the previous or this study. Recent studies have shown that the action of gut microbiota in physiological muscle function and systemic metabolism was priceless (19, 20, 25). Therefore, gut microbiota diversity was assessed in the cecum and colon digesta by 16s rDNA sequencing of microorganisms. We observed that high ammonia exposure elevated the alpha diversity, indicated by the indexes of Sobs, Shannon, ACE, or Chao, and induced the transition of microbiota composition recommended by beta diversity in the cecum and colon. Among that, the explanation of microbial shift provided by the R value of PCoA suggested that the cecal microbiota was more sensitive to aerial ammonia, which was consistent with the more remarkable alteration of microbiota in the cecum than that in the colon under the condition of ammonia exposure. Gut dysbiosis happens when the diversity, constitution, and functions of the gut microbiota are disturbed, negatively affecting an individual, for example, through interferece with intestinal homeostasis and disrupting immune response (26). The ratio of two main dominant phyla, Firmicutes and Bacteroidetes, displays a noticeable transition (decreased Firmicutes and increased Bacteroidetes) in patients with inflammatory bowel disease (27). In this study, greater abundance of Bacteroidetes and unchanged Firmicutes in the cecum were observed after ammonia exposure, which indicated that the lower ratio of Firmicutes/Bacteroidetes might contribute to inappropriate immune activation.

Specific bacteria (e.g., *Akkermansia, Alloprevotella, Lachnospira, Clostridium_sensu_stricto_1, Negativibacillus, Terrisporobacter*, and *Lactobacillus*) were identified to be enormously influenced by aerial ammonia exposure of the cecum or colon. For instance, *Akkermansia*, a commonly accepted beneficial inhabitant of the human microbiota, is crucial for fatty acid metabolism, which is conversely correlated with increased inflammation and reduction in patients with obesity or fatty liver disease (28). *Akkermansia* and its outer membrane protein AMUC_1100 could bind to a receptor on the membrane of intestinal epithelial cells and affect the downstream immune regulatory pathway, reducing inflammation response and LPS, regulating lipid and glucose metabolism (29, 30). Depletion of *Akkermansia* induced by ammonia exposure was observed, which might be a vital reason for metabolic disorder or inflammation after aerial ammonia exposure. Studies reported that the relatively great abundance of *Alloprevotella* and *Lachnospiraceae* was presented in the high-salt treatment, which could cause a metabolic disturbance, raising the risk of chronic disorders, tumors, and cardiovascular disease (31, 32). Although *Lachnospira* was well-described as a beneficial inhabitant for human and animal by fermenting fibers, thereby producing SCFA, it has been reported that aflatoxin B1 exposure induced a dramatic increment of *Lachnospira* (33), as *Lachnospira* was positively correlated with diarrhea in weaning pigs (34). All those reports indicated that *Alloprevotella* and *Lachnospira* might be potential pathogens under specific conditions. The data demonstrated that increased gut microbiota, *Alloprevotella* and *Lachnospira* might not be a beneficial signal for human and animal health under the condition of aerial ammonia exposure. Earlier research reported that the colonization of *Clostridium_sensu_stricto_1* could improve the aggregation of T cells regulated by CD41 in the colon of sterile mice (35) and strengthen the resistance of infant gut bacteria by hindering the colonization of pathogenic microbiota (36), which might explain the decrement of *Clostridium_sensu_stricto_1* induced by ammonia exposure in this study. Pathogenic bacteria *Negativibacillus* associated with gut dysbiosis or pediatric Crohn's disease (37, 38) was dramatically increased in this research after ammonia exposure. The results on specific different genera caused by ammonia exposure of the hindgut were quite different from those of the previous study on the small intestine (11), which is probably due to undigested or unabsorbed nutrients (induced by ammonia exposure in the small intestine) from the small intestine to enter the hindgut, interacting with ammonia exposure of the hindgut. Confusingly, *Lactobacillus*, identified as beneficial bacteria by numerous studies, was lifted in the intestine of ammonia-exposed pigs accompanied with metabolism disorder, especially lipid metabolism in skeletal muscle. Even so, a study still illustrated that a high-fat diet induced gut microbiota alteration (increased *Lactobacillus* and *Turicibacter*) in castrated mice and male androgen receptor knockout mice, causing metabolic disorder (39). Besides, Anhe et al. (40) found that camu camu (*Myrciaria dubia*) increased *Akkermansia* and decreased *Lactobacillus*, which increased heat production and made mice maintain metabolic homeostasis under the condition of high-fat and high-sugar intake. Therefore, the role of *Lactobacillus* raised by ammonia exposure needs to be further explored.

Microbial metabolites, closely related to the composition of intestinal microbiota, are incredibly crucial for the systemic metabolism and occurrence of metabolic diseases (21, 41, 42), and the BA profile, a kind of very vital microbial metabolites, was further analyzed in this research after ammonia exposure. The results demonstrated that the altered tendency of BA in the hindgut and serum was entirely dissimilar, which suggested that ammonia exposure enhanced the resorption of BAs, especially PBAs, in the ileum, causing a reduction in the PBA level entering the hindgut accompanied by decrement in SBAs produced by microbiota with PBA as substrate in the hindgut. The increment in BA resorption induced by ammonia exposure was consistent with the enhanced BA transport in the ileum after chronic heat exposure, and the serum THDCA level also declined in ammonia-exposed or heat-stressed pigs (43). However, heat stress had little effect on BA and BA-related bacteria in the hindgut, while ammonia exposure altered the hindgut BA profiles by disturbing the hindgut microbiota. The DCA and LCA, the most abundant metabolites in the gut microbiome, modify host energy and metabolism as well as gut barrier and inflammation (21, 42). For instance, the DCA and LCA, whose production is closely related to *Clostridium scindens*, are favorable for maintaining gut barrier integrity or accelerating intestinal crypt regeneration and wound repair *via* the farnesoid X receptor (FXR) and also detrimental for pathogen colonization (21, 44). We observed that the SBA content notably dropped with the relative abundance of *Clostridium_sensu_stricto_1* decreasing in the cecum after ammonia exposure, which might cause detrimental impacts on the intestinal barrier or inflammation and could lead to lipid or glucose metabolism disorder. Besides, the decreased SBA was affected by genera *Terrisporobacter, Clostridium_sensu_stricto_1, Turicibacter*, and *Lactobacillus* indicated by the RDA analysis. The increment in serum BA was essential in whole systemic metabolism after ammonia exposure in this research. Takeda G protein-coupled receptor 5 (TGR5), the plasma membrane-bound G protein-coupled receptor ubiquitously expressed with high expression in skeletal muscle, white adipose tissues, and gut (45, 46) is another crucial BA-responsive receptor that participated in host metabolism. The SBA (including LCA and DCA) is the primary ligand to activate TGR5 (47), and then the activation of TGR5 can regulate host metabolism through the mTOR pathway (48). The previous study showed that high ammonia exposure activated the mTOR-p70s6k pathway, leading to lipid metabolic disorder in skeletal muscle, which might be due to the increment in serums HCA, LCA, and HDCA induced by ammonia exposure in this study. Simultaneously, increased serum BA (including HCA, LCA, and HDCA) caused by ammonia exposure was affected by genera *Clostridium_sensu_stricto_1, Terrisporobacter, Alloprevotella, norank_f_Muribaculaceae*, and *Lactobacillus* indicated by the network analysis or Mantel's correlation in the research. The function of serum low abundance BA, such as TDCA or THDCA, being altered by ammonia exposure to host metabolism needs further *in vitro* trial investigation.

Short-chain fatty acid in the hindgut and specific serum AA (including BCAA and aromatic AA), two other kinds of crucial metabolites interacting with the gut microbiota to affect intestinal health, inflammation, and systemic metabolism (49, 50), was also measured in this study after ammonia exposure. Aerial ammonia exposure increased the content of hindgut acetate in this study. In addition, acetic-producing bacteria *Lactobacillus* increased, and the main reason for increased acetate in the hindgut was that ammonia exposure caused insufficient digestion and absorption of nutrients in pigs, resulting in starch and small molecular sugars entering the hindgut and fermenting to produce more acetate. Multitudinous studies have confirmed that ammonia exposure reduces animal growth performance (7, 51, 52). Despite lower values of BW on day 21 and day 25 in ammonia-exposed pigs, it did not reach the statistical level, suggesting that the adverse effect of impaired digestion and absorption of nutrients in the small intestine induced by ammonia exposure on growth performance of pigs could not be seen in the first 25 days. Simultaneously, undigested or unabsorbed nutrients entering the hindgut to provide substrates for hindgut microbiota might also be crucial for the increased microbial diversity in the hindgut. Studies confirmed that microbial-derived acetate takes part in the increment of fatty acid *de novo* synthesis and antibiotic-treated mice manifest decrement of fatty acid *de novo* synthesis (53), which might partly explain the results, namely disordered serum lipid-related metabolites and a decrease in fatty acid oxidation or an increase in fatty acid synthesis in skeletal muscle after ammonia exposure. Besides, the content of isobutyrate and isovalerate was declined with SCFA-related genera (*Terrisporobacter, Turicibacter*, and *Clostridium_sensu_stricto_1*) altered in this study after ammonia exposure. BCAAs are partly produced and metabolized by the gut microbiota, and increased BCAA ciculation with dysbiotic intestinal microbiota is closely associated with metabolic disorders, such as insulin resistance (54, 55). The research found that increased BCAA circulation induced by ammonia exposure was partly explained by altered genera *Ralstonia, Akkermansia, unclassified_p_Firmicutes, Terrisporbacter*, and *Fammilly_XIII_AD3011_group*, and another reason for increased BCAA circulation was the inhibition of BCAA catabolism in skeletal muscle and the liver (unpublished data). Although extremely crucial aromatic AA tryptophan was unaffected by ammonia exposure, serums Phe and Tyr were dramatically increased, which was closely related to altered genera *Ralstonia, Akkermansia, unclassified_p_Firmicutes, Terrisporbacter, Fammilly_XIII_AD3011_group, Lactobacillus*, and *norank_f_Muribaculaceae*.

## Conclusion

In conclusion, high ammonia exposure damaged the bacterial composition and community structure of hindgut microbiota in growing pigs, increasing the presence of potentially pathogenic bacteria, such as *Negativibacillus, Alloprevotella*, or *Lachnospira*, and decreasing the presence of beneficial bacteria, such as *Akkermansia* or *Clostridium_sensu_stricto_1*. The dysbiotic microbiota caused the increment in hindgut acetate and decrement in cecal BAs (mainly including CA, HCA, DCA, LCA, and HDCA) and led to the increment in serum BAs (primarily involving HCA, LCA, and HDCA), BCAA, or aromatic AA, which might serve as signaling molecules to interact with host metabolism (Table 1). This study provides a novel sight to partly explain the metabolism disorder or tissue injury induced by atmospheric ammonia exposure.

**Table 1:** Summary of the altered metabolites from host serum or hindgut chyme after high ammonia exposure.

		**Ammonia exposure**
Serum AA (16)	BCAA	Ile ↑, Leu ↑, Val ↑
	Aromatic AA	Phe ↑, Tyr ↑
Serum BA	Free-PBA	HCA ↑
	Free-SBA	LCA ↑, HDCA ↑
	TCBA	TDCA ↓, THDCA ↓
		SBA ↑
Serum lipid-related metabolites		TG ↑ (16), ApoB ↑ (16), HDL-C ↓
Serum lipid-related hormones		Leptin ↓
Hindgut SCFA	Cecal SCFA	Total SCFA ↑, acetate ↑, isobutyrate ↓, isovalerate ↓
	Colonic SCFA	Acetate ↑, isobutyrate ↓, isovalerate ↓
Cecal BA	Free-PBA	CA ↓, HCA ↓
	Free-SBA	DCA ↓, LCA ↓, HDCA ↓
	GCBA	GCDCA ↑
		Total BA ↓, SBA↓, GCBA↑

*↓ Indicates decrease and ↑ indicates increase*.

## Data Availability Statement

The data used to support the findings of this study are available from the corresponding author upon request. The raw data on cecal or colonic digesta microbiota of pigs were deposited in NCBI's Sequence Read Archive (SRA) database and accessible through SRA accession number: PRJNA718995.

## Ethics Statement

The animal study was reviewed and approved by the Experimental Animal Welfare and Ethical Committee of Institute of Animal Science of Chinese Academy of Agricultural Sciences.

## Author Contributions

ST, RZ, and HZ designed the research. ST, RZ, CY, and DS conducted the research. ST and LC analyzed the data. ST and HZ wrote the study and had primary responsibility for the final content. LL and JX provided the animals and expertise. All authors read and approved the final manuscript.

## Conflict of Interest

The authors declare that the research was conducted in the absence of any commercial or financial relationships that could be construed as a potential conflict of interest.

## Abbreviations

AA: amino acid
ALT: alanine aminotransferase
AST: aspartate aminotransferase
BA: bile acid
BCAA: branched-chain amino acid
BW: body weight
BWG: body weight gain
CA: cholic acid
CDCA: chenodeoxycholic acid
DCA: deoxycholic acid
FXR: farnesoid X receptor
GCA: glycol-cholic acid
GCBA: glycine-conjugated bile acid
GCDCA: glycol-chenodeoxycholic acid
GHCA: glycol-hyocholic acid
GUDCA: glycol-ursodeoxycholic acid
HCA: hyocholic acid
HDCA: hyodeoxycholic acid
HDL-C: high-density lipoprotein cholesterol
Ile: isoleucine
LCA: lithocholic acid
LDL-C: low-density lipoprotein cholesterol
Leu: leucine
LPS: lipopolysaccharide
MyHC: myosin heavy chain
NH_3_: ammonia
OTU: operational taxonomic unit
PBA: primary bile acid
PCoA: principal coordinate analysis
Phe: phenylalanine
RDA: redundancy analysis
SBA: secondary bile acid
SCFA: short-chain fatty acid
TBA: total bile acid
TC: total cholesterol
TCA: tauro-cholic acid
TCBA: taurine-conjugated bile acid
TCDCA: tauro-chenodeoxycholic acid
TDCA: tauro-deoxycholic acid
TG: total triglycerides
TGR5: takeda G protein-coupled receptor 5
THDCA: tauro-hyodeoxycholic acid
TLCA: tauro-lithocholic acid
TUDCA: tauro-ursodeoxycholic acid
Tyr: tyrosine
UDCA: ursodeoxycholic acid
Val: valine
VLDL: very low density lipoprotein.
