# Supplementary material for: Exposure to High Aerial Ammonia Causes Hindgut Dysbiotic Microbiota and Alterations of Microbiota-Derived Metabolites in Growing Pigs
Source: Front Nutr. 2021 Jun 11;8:689818. doi: 10.3389/fnut.2021.689818 (PMC8231926; doi:10.3389/fnut.2021.689818)

**Supplementary tables and figures**

**Table S1** Composition and nutrient levels of the basal diet (air-dry basis)

| **Item** | **Percentage, %** | **Item** | **Percentage** |
| --- | --- | --- | --- |
| Ingredients |  | Nutrient level^b^ |  |
| Corn | 65.00 | Digestible energy, Mcal/kg | 3.15 |
| Wheat bran | 5.00 | Crude protein, % | 18.50 |
| Soybean meal | 26.00 | Lysine, % | 1.00 |
| Premix ^a^ | 4.00 | Threonine, % | 0.74 |
| Total | 100.00 | Calcium, % | 0.75 |
|  |  | Total phosphorus, % | 0.65 |
|  |  | Available phosphorus, % | 0.40 |

^a^ The premix provided the following per kg of the diet: 100000 IU vitamin A, 50000 IU vitamin D3, 400 mg vitamin E, 30 mg vitamin K3, 45 mg vitamin B1, 100 mg vitamin B2, 70 mg vitamin B6, 0.35 mg vitamin B12, 550 mg nicotinic acid, 450 mg pantothenic acid, 18 mg folic acid, 5 mg biotin, 15 g choline chloride, 1 g Fe, 0.2 g Cu, 1 g Zn, 0.5 g Mn, 3.5 mg I, 2.5 mg Se, 10% Ca, 2.5% total phosphorus, 10% water, 3% Lysine.

^b^ Estimated using the NRC (2012) individual dietary ingredients.

**Table S2** Details of different metabolite from host serum in control or ammonia pigs

|  |  |  | **Groups** | | ***p*-value** |
| --- | --- | --- | --- | --- | --- |
|  |  |  | **Control** | **Ammonia** |  |
| **AA (nmol/mL) (Tang et al., 2020)** | BCAA | Isoleucine | 82.48±5.28b | 110.88±4.01a | 0.0016 |
|  |  | Leucine | 146.57±5.51b | 207.00±6.91a | <0.0001 |
|  |  | Valine | 187.08±9.37b | 232.97±8.55a | 0.0047 |
|  | Aromatic AA | Phenylalanine | 67.88±3.51b | 96.88±3.63a | 0.0002 |
|  |  | Tyrosine | 59.15±2.32b | 108.56±6.63a | <0.0001 |
|  |  | Tryptopha | 42.78±3.07 | 48.43±3.33 | 0.2405 |
| **BA (ng/mL)** | Free-PBA | CA | 26.32±1.75 | 26.58±2.03 | 0.9252 |
|  |  | CDCA | 4458.65±1079.42 | 6840.68±1526.63 | 0.2315 |
|  |  | HCA | 1118.22±197.51 | 1947.34±338.65 | 0.0605 |
|  | Free-SBA | DCA | 35.81±2.08 | 34.28±1.42 | 0.5554 |
|  |  | UDCA | 145.55±55.20 | 159.03±37.95 | 0.8445 |
|  |  | LCA | 293.82±37.10 | 402.44±41.60 | 0.0799 |
|  |  | HDCA | 9845.78±2715.21 | 17312.51±2790.99 | 0.0841 |
|  | GCBA | GCA | 29.83±1.73 | 32.64±1.69 | 0.2723 |
|  |  | GDCA | 14.78±1.68 | 12.83±1.35 | 0.3844 |
|  |  | GCDCA | 1124.43±222.00 | 1226.71±117.14 | 0.6922 |
|  |  | GUDCA | 29.86±5.30 | 31.85±2.92 | 0.7492 |
|  |  | GHCA | 22.11±1.36 | 24.02±0.98 | 0.2818 |
|  | TCBA | TCA | 49.47±5.15 | 63.51±14.19 | 0.3743 |
|  |  | TDCA | 26.80±0.56 | 25.63±0.20 | 0.0751 |
|  |  | TCDCA | 432.67±78.81 | 365.10±73.22 | 0.5440 |
|  |  | TUDCA | 313.76±85.72 | 446.06±61.74 | 0.2389 |
|  |  | THDCA | 30.02±0.36a | 28.66±0.11b | 0.0050 |
|  |  | TLCA | 26.55±1.60 | 29.48±2.20 | 0.3078 |
|  | Total BA |  | 18024.45±4087.66 | 29009.32±4613.48 | 0.1051 |
|  | PBA |  | 7261.71±1303.78 | 10526.57±1921.37 | 0.1900 |
|  | SBA |  | 10762.74±2850.76 | 18482.75±2891.70 | 0.0865 |
|  | GCBA |  | 1221.02±225.51 | 1328.04±121.73 | 0.6851 |
|  | TCBA |  | 879.29±142.10 | 958.43±93.15 | 0.6513 |
|  | PBA/SBA |  | 0.58±0.05 | 0.49±0.06 | 0.1321 |
| **Lipid-related metabolites** |  | TC (mmol/L) (Tang et al., 2020) | 2.21±0.11 | 2.12±0.09 | 0.5502 |
|  |  | TG (mmol/L) (Tang et al., 2020) | 0.39±0.03b | 0.61±0.08a | 0.0294 |
|  |  | ApoAI (g/L) (Tang et al., 2020) | 0.55±0.01 | 0.59±0.03 | 0.2361 |
|  |  | ApoB (g/L) (Tang et al., 2020) | 0.48±0.04b | 0.67±0.03a | 0.0061 |
|  |  | HDL-C (mmol/L) | 0.97±0.03a | 0.80±0.02b | 0.0006 |
|  |  | LDL-C (mmol/L) | 1.04±0.10 | 1.08±0.07 | 0.6975 |
|  |  | VDL (mmol/L) | 0.21±0.01 | 0.25±0.04 | 0.4328 |
| **Lipid-related hormones** |  | Insulin (ng/mL) (Tang et al., 2020) | 10.74±0.69 | 11.85±0.60 | 0.2521 |
|  |  | Leptin (pg/mL) | 1078.30±33.55 | 1000.74±24.09 | 0.0898 |
|  |  | Adiponectin (ng/mL) | 1807.62±50.68 | 1721.57±72.78 | 0.3548 |
|  |  | ALT (U/L) | 53.95±3.69 | 64.63±7.07 | 0.2100 |
|  |  | AST (U/L) | 34.98±4.45 | 43.37±7.63 | 0.3651 |

All data are expressed as mean ± SE (n = 6 pigs per group). In the same row, value with different small letter superscripts means a significant difference (*p* < 0.05). The same as below.

**Table S3** Details of different metabolite from hindgut chyme in control or ammonia pigs

|  |  |  | **Groups** | | ***p*-value** |
| --- | --- | --- | --- | --- | --- |
|  |  |  | **Control** | **Ammonia** |  |
| **SCFA (umol/g wet weight)** | Cecum | Acetate | 62.18±5.09b | 76.86±2.19a | 0.0243 |
|  |  | Propionate | 25.46±2.82 | 31.68±2.31 | 0.1188 |
|  |  | Isobutyrate | 1.51±0.09a | 0.84±0.07b | 0.0002 |
|  |  | Butyrate | 10.83±2.56 | 13.32±1.24 | 0.4016 |
|  |  | Isovalerate | 2.00±0.12a | 1.02±0.08b | 0.0001 |
|  |  | Valerate | 2.02±0.12 | 1.81±0.23 | 0.4278 |
|  |  | Acetate/Propionate | 2.53±0.27 | 2.47±0.14 | 0.8405 |
|  |  | Total SCFA | 104.00±9.01b | 125.53±5.23a | 0.0657 |
|  | Colon | Acetate | 79.73±2.82 | 91.75±5.00 | 0.0624 |
|  |  | Propionate | 30.09±3.46 | 32.99±4.00 | 0.5923 |
|  |  | Isobutyrate | 3.20±0.21a | 2.41±0.14b | 0.0115 |
|  |  | Butyrate | 20.38±2.93 | 20.68±2.42 | 0.9391 |
|  |  | Isovalerate | 5.27±0.35a | 3.65±0.24b | 0.0035 |
|  |  | Valerate | 6.18±0.58 | 5.20±0.36 | 0.1792 |
|  |  | Acetate/Propionate | 2.77±0.22 | 2.92±0.27 | 0.6830 |
|  |  | Total SCFA | 144.84±9.97 | 156.68±10.44 | 0.4312 |
| **Cecal BA (ng/mg lyophilized weight)** | Free-PBA | CA | 10.22±2.36 | 4.91±0.81 | 0.0586 |
|  |  | CDCA | 237.91±43.21 | 246.84±21.93 | 0.8575 |
|  |  | HCA | 566.88±139.95 | 268.27±46.27 | 0.0703 |
|  | Free-SBA | DCA | 13.31±3.76a | 3.47±0.34b | 0.0262 |
|  |  | UDCA | 118.51±47.29 | 59.01±17.08 | 0.2640 |
|  |  | LCA | 2452.04±610.47 | 1273.68±97.52 | 0.0858 |
|  |  | HDCA | 3941.98±324.94a | 3038.50±218.34b | 0.0437 |
|  | GCBA | GCA | 0.44±0.11 | 0.69±0.15 | 0.1892 |
|  |  | GDCA | 0.47±0.19 | 0.19±0.08 | 0.1979 |
|  |  | GCDCA | 51.70±12.70b | 133.28±23.81a | 0.0128 |
|  |  | GUDCA | 0.66±0.19 | 0.98±0.24 | 0.3101 |
|  |  | GHCA | 0.55±0.21 | 0.63±0.13 | 0.7604 |
|  | TCBA | TCA | 74.03±18.19 | 43.08±5.92 | 0.1369 |
|  |  | TDCA | 1.22±0.59 | 0.84±0.34 | 0.5871 |
|  |  | TCDCA | 766.97±252.35 | 580.16±211.25 | 0.5828 |
|  |  | TUDCA | 52.56±9.38 | 53.18±18.96 | 0.9770 |
|  |  | THDCA | 0.67±0.46 | 0.51±0.16 | 0.7523 |
|  |  | TLCA | 2.69±0.68 | 2.81±0.75 | 0.9061 |
|  | Total BA |  | 8292.81±1129.32 | 5711.03±350.85 | 0.0540 |
|  | PBA |  | 1708.70±352.28 | 1277.86±213.96 | 0.3205 |
|  | SBA |  | 6584.10±946.75 | 4433.17±322.10 | 0.0570 |
|  | GCBA |  | 53.82±12.82b | 135.77±24.35a | 0.0139 |
|  | TCBA |  | 898.14±269.93 | 680.58±225.61 | 0.5501 |

**
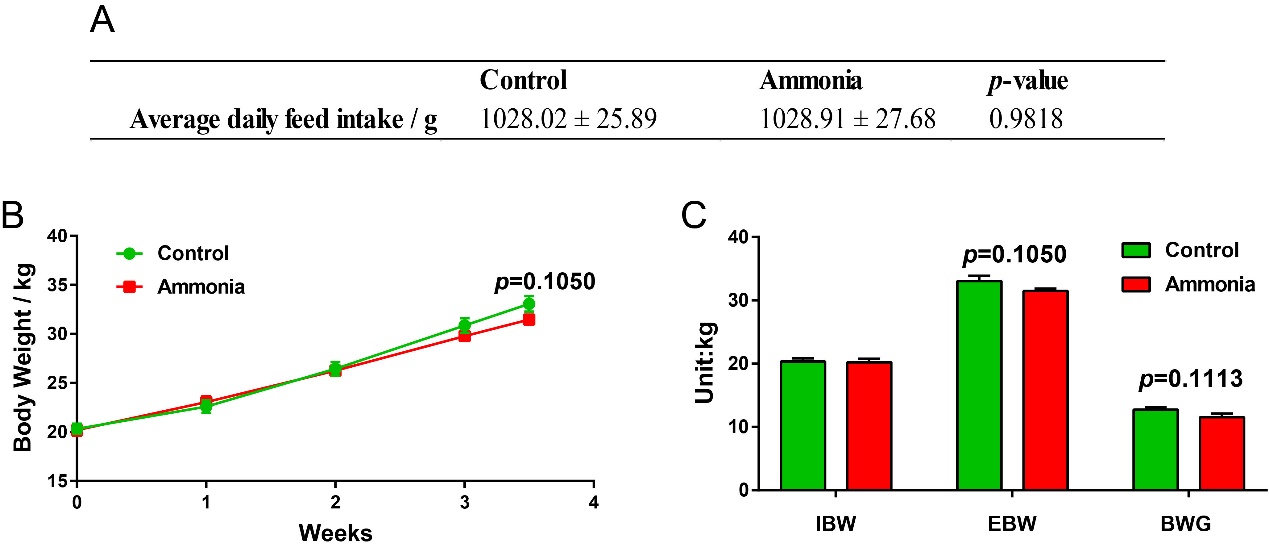
Figure S1.** Growth performance of pigs after high atmospheric ammonia exposure (Tang et al., 2020). Data are expressed as mean ± SE (n = 6 per group). IBW, initial body weight; EBW, end body weight; BWG, body weight gain.

**Figure S2.** The rarefaction curves. Rarefaction curves of Sobs index (A or D), Shannon index (B or E) and Chao index (C or F) on OTU level for each sample of cecal or colonic digesta.
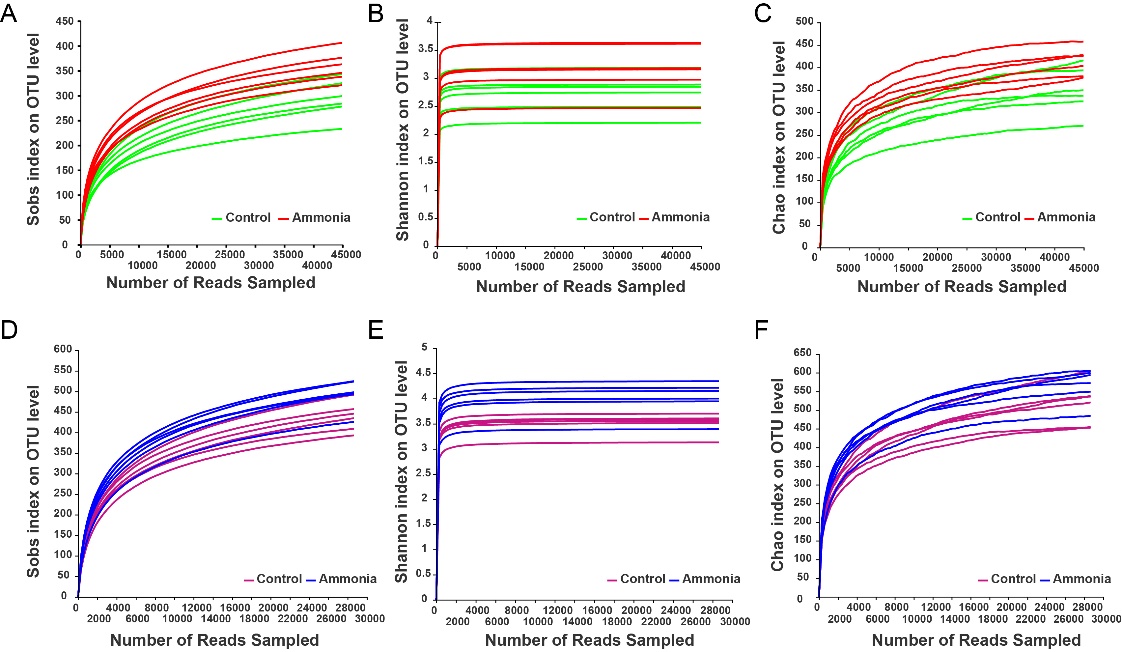

Supplement: Supplementary file 1 [file Data_Sheet_1.docx]
